# First Human Leucocyte Antigen (HLA) Response and Safety Evaluation of Fibrous Demineralized Bone Matrix in a Critical Size Femoral Defect Model of the Sprague-Dawley Rat

**DOI:** 10.3390/ma13143120

**Published:** 2020-07-13

**Authors:** Nicolas Söhling, Maximilian Leiblein, Alexander Schaible, Maren Janko, Joachim Schwäble, Christian Seidl, Jan C. Brune, Christoph Nau, Ingo Marzi, Dirk Henrich, René D. Verboket

**Affiliations:** 1 Department of Trauma, Hand and Reconstructive Surgery, Goethe University Frankfurt, 60590 Frankfurt am Main, Germany; Nicolas.Soehling@kgu.de (N.S.); maximilian.leiblein@kgu.de (M.L.); alexanderschaiblex@gmail.com (A.S.); maren.janko@kgu.de (M.J.); Christoph.Nau@kgu.de (C.N.); marzi@trauma.uni-frankfurt.de (I.M.); d.henrich@trauma.uni-frankfurt.de (D.H.); 2 Institute for Transfusion Medicine and Immunohematology, Goethe University Hospital Medical School, German Red Cross Blood Donor Service, 60528 Frankfurt, Germany; j.schwaeble@blutspende.de (J.S.); c.seidl@blutspende.de (C.S.); 3 German Institute for Cell- and Tissue Replacement (DIZG, gemeinnützige GmbH), 12555 Berlin, Germany; j_brune@dizg.de

**Keywords:** demineralized bone matrix, rat femur critical size defect

## Abstract

Treatment of large bone defects is one of the great challenges in contemporary orthopedic and traumatic surgery. Grafts are necessary to support bone healing. A well-established allograft is demineralized bone matrix (DBM) prepared from donated human bone tissue. In this study, a fibrous demineralized bone matrix (f-DBM) with a high surface-to-volume ratio has been analyzed for toxicity and immunogenicity. f-DBM was transplanted to a 5-mm, plate-stabilized, femoral critical-size-bone-defect in Sprague-Dawley (SD)-rats. Healthy animals were used as controls. After two months histology, hematological analyses, immunogenicity as well as serum biochemistry were performed. Evaluation of free radical release and hematological and biochemical analyses showed no significant differences between the control group and recipients of f-DBM. Histologically, there was no evidence of damage to liver and kidney and good bone healing was observed in the f-DBM group. Reactivity against human HLA class I and class II antigens was detected with mostly low fluorescence values both in the serum of untreated and treated animals, reflecting rather a background reaction. Taken together, these results provide evidence for no systemic toxicity and the first proof of no basic immunogenic reaction to bone allograft and no sensitization of the recipient.

## 1. Introduction

Treatment of large bone defects is one of the great challenges in contemporary orthopedic and traumatic surgery. Traditionally, autologous bone grafts, vascularized and nonvascularized, have been used to restore damaged bone [1]. Their tissue-compatibility and osteogenic properties (mechanically stable, osteoinductive and -conductive, promoting vascularization) seem to provide the ideal bone void filler. However, due to donor site morbidity and limited availability, alternative approaches are necessary [2,3,4,5]. New developments have to compete with autologous grafts. Numerous attempts have been made and there are several products available now for clinical daily routine—ranging from human allografts to xenograft and synthetic bone substitute materials [6,7].

A well-established allograft is demineralized bone matrix (DBM), prepared from donated human bone [8,9,10]. Once harvested, the bone can be processed through different methods, including physical debridement, ultrasonic washing, treatment with ethylene oxide, antibiotic washing, and gamma irradiation or peracetic acid treatment for spore elimination [11]. Immunogenic capacities are massively reduced by processing. Host versus graft reactions as well as disease transmission are very rarely reported for fresh grafts and never reported for sterilized allografts. Cellular infiltration around the implantation sites as an indication of acute inflammation has not been observed so far. However, the long-term course in terms of T-cell mediated chronic rejection is being discussed. A prerequisite for this is the presence of HLA proteins in the allograft [12]. Fretwurst et al. were able to detect HLA proteins in commercially available DBM samples [13]. A specific immune response with specific antibodies to these proteins has not yet been demonstrated. However, Strong et al. detected antibodies against HLA complexes in fresh frozen bone transplanted patients [14]. In contrast to frozen or freeze-dried bone, the final mineral fraction is reduced to an amount of 1–6 percent by extraction [15]. Microscopically, the remaining tissue shows a trabecular structure, consisting of collagen (mainly type 1), noncollagenous proteins and growth factors [15]. Available as granules, chips, fibers or paste (with additives) dry DBM holds the biological drivers for osteoconductive and osteoinductive properties [15,16,17,18]. Interconnected pores and the microstructured surface offer good conditions for cell adhesion, cell ingrowth, vascularization and connective tissue proliferation (osteoconductivity) [19]. Osteoinductive properties are attributed to growth factors, especially BMP (bone morphogenic protein)-2, with decreasing calcium levels leading to a decomplexation of growth factors and proteins [17,20,21]. Early data in mice by Urist et al. confirmed expected ectopic osteogenic properties in animal experiments [21]. DBM granules implanted subcutaneously were able to induce bone formation. Rehydration is necessary before use. The ready-to-use DBM has a putty-like consistency and bears low mechanical strength. So DBM is mainly used clinically in areas where only small defects need to be bridged with only low mechanical stress. Examples of successful applications are the support of osseointegration of dental implants, but also to support arthrodesis procedures on joints. Even in human spine surgery the results were promising [22,23,24,25,26,27]. But clinical application in large bone defect is rare and mainly restricted to experimental approaches since DBM is easy to place in the bony gap, but a challenge to keep it there. Hovathy et al. successfully treated critical-size bone defects in rats with pure DBM [28]. Janko et al. showed accelerated bone defect healing in rat critical size femur defects by combination of DBM with BMC [29].

Numerous DBM preparations are commercially available, all with significant differences in the preparation process. A recent development is DBM fibers that possess a structural coherence even after rehydration, facilitating the positioning of the DBM inside a defect in contrast to DBM putty. In addition, the greater surface-to-volume ratio compared to DBM chips and blocks is expected to improve cell adhesion, cell loading capacity and growth factor access.

In this study we examined toxicity, immunogenic behavior and osteogenicity two months after a fibrous DBM (f-DBM) preparation was transplanted into a plate-stabilized, femoral critical-size-bone-defect of 5 mm in Sprague-Dawley (SD) rats. As a control group, healthy animals without surgery were selected to represent the physiological normal status. We hypothesize that the application of f-DBM even in a foreign organism is safe and try to demonstrate this for the first time in rats using the highly sensitive Luminex method.

## 2. Materials and Methods

### 2.1. Preparation of Fibrous Scaffold

Tissue grafts in Germany are regulated as medicinal products under the German Medicinal Products Act (Arzneimittelgesetz, AMG, federal law, Federal Republic of Germany). All tissues are acquired from nonprofit tissue donation organizations after informed consent and according to German AMG and TPG (Transplantation Act, Transplantationsgesetz, federal law, Federal Republic of Germany) regulations. Stringent serological screening is then followed by tissue preparation under controlled CNC- (Computerized Numerical Control) mill-conditions, the bone is clamped into the CNC machine and cut into fibrous tissue scaffold by a milling head, resulting in a cortical and a cancellous bone fraction. Subsequently the tissue is partially demineralized and subjected to a validated sterilization process as described previously [30]. The fibrous tissue scaffold is then preserved by lyophilization and packaged.

### 2.2. Ethics

All animal experiments were performed in accordance with the institutional animal care and oversight committee (Project FK/1075, Regierungspräsidium Darmstadt, Germany), in accordance with German law. All surgery was performed under general anesthesia, administered intraperitoneally as a mixture of Ketavet and Rompun. All efforts were made to minimize suffering. The animals were sacrificed with an overdose of intraperitoneal pentobarbital (150 µg/kg).

### 2.3. Animal Care and Defect Surgery

For the critical-sized defect model, eleven male SD rats were used (7 f-DBM, 4 control group without intervention), which were 8 weeks old and weighed approximately 250–300 g. The animals were purchased from Janvier, France and four animals per cage were housed together with food and water ad libitum. The temperature was regulated (15–21 °C) in an air flow- and light-controlled (14 h per day, 10 h night) environment.

A general anesthesia with 2 ml of a mixture of Ketavet (70 mg/kg) and Rompun (10 mg/kg) was administered intraperitoneally. Under aseptic conditions, the right rat femur was dissected. A five-hole plate was prepared (Miniplate Lockingplate LCP Compact Hand 1.5 straight, Depuy-Synthes, Dubendorf, Switzerland) and positioned on the femur and secured in place with four 1,3 mm cortical screws (CompactHand, Synthes), leaving the middle hole free. Using a Gigli saw a 5 mm defect of the femur under the free middle hole of the plate was created. A 5 mm defect in a rat femur was shown to be a critical size defect in previous studies [31]. The fibrous scaffold was implanted into the segmental defect and the wound was closed in two layers with continuous subcutaneous stitches using a 4/0-monofilament nylon suture. Animals were randomly assigned to either the treatment or the control group. As postoperative analgesia, the animals received 2.6 mg/kg Carprofen s.c. on operation and on the first following day and overlapping 2.5 mg/100mL Tramadol in the drinking water as pain treatment over the following five days. The rats were housed for twelve weeks under standard conditions and nutrition.

**Figure 1:**
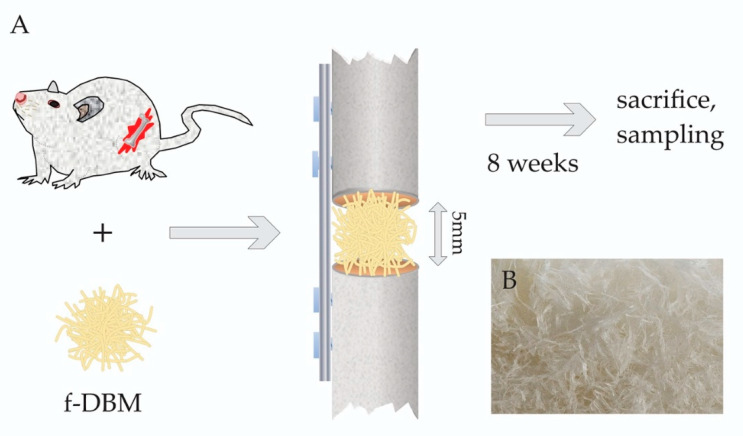
(**A**): Schematic of the experimental set-up: Implantation of fibrous-demineralized bone matrix (f-DBM) in 5 mm critical size femoral defects in Sprague-Dawley rats, which were sacrificed after 8 weeks. (**B)**: Macroscopic image of the fibrous tissue scaffold.

The animals were sacrificed eight weeks later by inhalation of anesthesia and intracardial pentobarbital injection (150 mg/kg) (Figure 1).

### 2.4. Blood Sampling, Femur and Internal Organ Collection

At the time of sacrifice, blood was collected after inhalation of anesthesia from the abdominal aorta in blood collection tubes for serum and plasma for hematological tests (ethylendiaminetetraacetic acid (EDTA))- and serum monovettes, Sarstedt, Nümbrecht, Germany). The liver, kidney and spleen were removed then weighed and fixed in 4% zinc-formalin (Thermo Scientific, Schwerte, Germany). The femoral bone was exposed, wrapped in gauze humidified with physiological NaCl-solution and stored at −80 °C until preparation for (immuno)histological examination. The explanted bones were examined regarding signs of infection or tumors and the firm fit of the implanted screws was checked.

### 2.5. Histology

Paraffin-embedded organ sections (5 mm) were stained with haematoxilin/eosin (HE) and chloracetate esterase (CAE). Femoral bones were decalcified over 7 days in a 10% Tris buffered EDTA-solution under continuous stirring. Afterwards the bones were embedded in paraffin and sections (5 mm) sagittal to the bone were taken and stained with HE and Orange G+ staining [32]. One slide per animal and organ was analyzed using light microscopy (Axioobserver Z1, Zeiss, Göttingen, Germany) at a magnification of 100× in combination with a computer-supported imaging picture analysis system (Axiovision 4.7, Zeiss, Göttingen, Germany). This was done by an independent observer blinded to the experimental group set up. The animal ID was assigned for evaluation and is kept constant throughout the document.

### 2.6. Bone Healing Score

The bone healing score was determined analogous to Han et al. [33] with the following addition. The bone marrow portion in the defect area was included as an additional category to take into account the formation of the bone marrow cavities during bone remodeling. Analogous to the formation of new bone, score values between 0 (corresponds to 0–10% of the remnant defect) and 10 (corresponds to 90–100% of the remnant defect) were applied. The remnant defect was defined as defect area minus newly formed bone tissue. Higher values indicate better bone healing.

### 2.7. Haematological Analysis

Hemoglobin concentration, red and white blood cell counts and subpopulation counts were measured by conventional blood analysis (AMP Accos 5110, AMEDA Labordiagnostik GmbH, Graz, Austria).

### 2.8. Serum Biochemistry

Glutamate oxaloacetate transaminase (GOT), glutamate pyruvate transaminase (GPT), alkaline phosphatase (ALP), creatinine, urea, glucose and total protein were quantified using the AMP Piccos II (AMEDA Labordiagnostik GmbH, Graz, Austria). Serum concentrations of NO-2, malondialdehyde (MDA), glutathione (GSH) and superoxide dismutase (SOD) activity were measured by colorimetric assay kits (Cayman Biomol GmbH, Hamburg, Germany) and evaluated using Infinite F50 (Tecan AG, Männedorf, Switzerland) and Magellan v 6.5 software (Tecan AG, Männedorf, Switzerland).

### 2.9. HLA Diagnostics

For detection of anti-human-HLA class I and class II antibodies in the sera of rats the LABScreen Single Antigen HLA Class I–Combi and the LABScreen Single Antigen HLA Class II Group 1 kits (OneLambda, West Hills, CA, USA) were used according to the manufacturer’s protocol, except that the polyethylene (PE)-labelled goat anti-human antibody from the kits was replaced by a PE-labelled goat anti-rat IgG antibody (CAT: 112-116-071, Jackson Laboratories, West Grove, PA, USA). Samples were measured with a LABScan3D (OneLambda, West Hills, CA, USA). The serum of an untreated rat served as a negative control. To generate a positive control, a rat anti-human HLA ABC antibody (CAT: MCA485G, Bio-Rad, Hercules, CA, USA) was diluted 1:100 in the serum of an untreated animal. Reactivity was considered positive when the normalized mean fluorescence intensity (MFI) was ≥ 1000.

### 2.10. Statistics

Results are presented as median (25% quartile/75% quartile) and nonparametric Kruskal–Wallis testing was applied, followed by a Bonferroni–Holm adjusted multiple Conover–Iman post-hoc analysis. A *p* value < 0.05 indicates significance. Statistics were calculated using the software Bias 11.09 (Epsilon-Verlag, Darmstadt, Germany). Correlations between parameters were analyzed using the Spearman Rank test. A *p* value < 0.05 indicates a significant correlation.

## 3. Results

### 3.1. No Difference in Survival Weight and Organ Function

All animals survived the 2 months follow-up with no signs of physical or neurological impairment. None of them showed abnormal behavior, nor signs of infection or delayed wound healing. Solely one animal (ID 9) that underwent surgery was excluded due to implant loosening.

**Figure 2:**
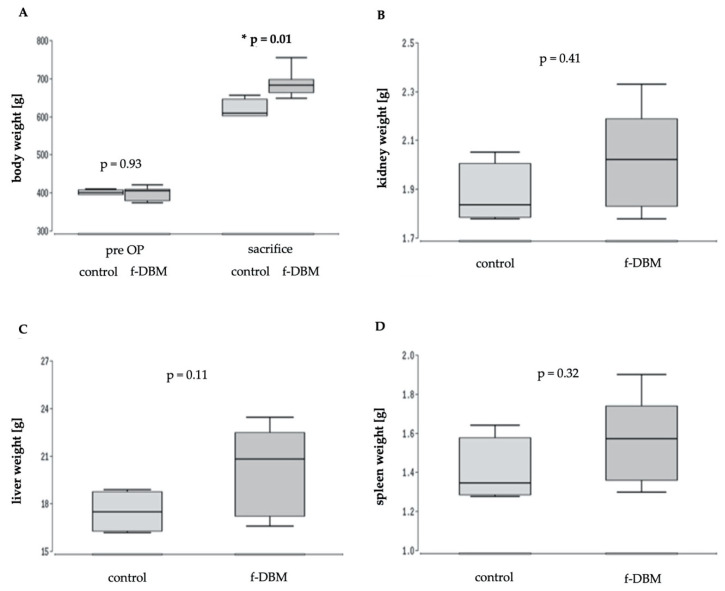
Weight gain in the two groups over the follow-up period. (**A**) Significant weight increase in both groups. Weight gain of selected organs at the time of sacrifice: (**B**) liver, (**C**) kidney, (**D**) spleen.

Starting with an almost identical weight (control: 400 g, f-DBM: 405 g, median values) both animal groups showed a statistically relevant increase of bodyweight over time. The final weight at the day of sacrifice was significantly higher in the treated animals than in the control group (control: 609 g, f-DBM: 682 g; median values; Figure 2A). Similar to the increase in body weight, a higher median weight of organs (liver, kidney, spleen) was found in the operated animals. However, the differences did not reach a level of significance (Figure 2B–D).

### 3.2. Histological Responses

#### Implantation Site

**Figure 3:**
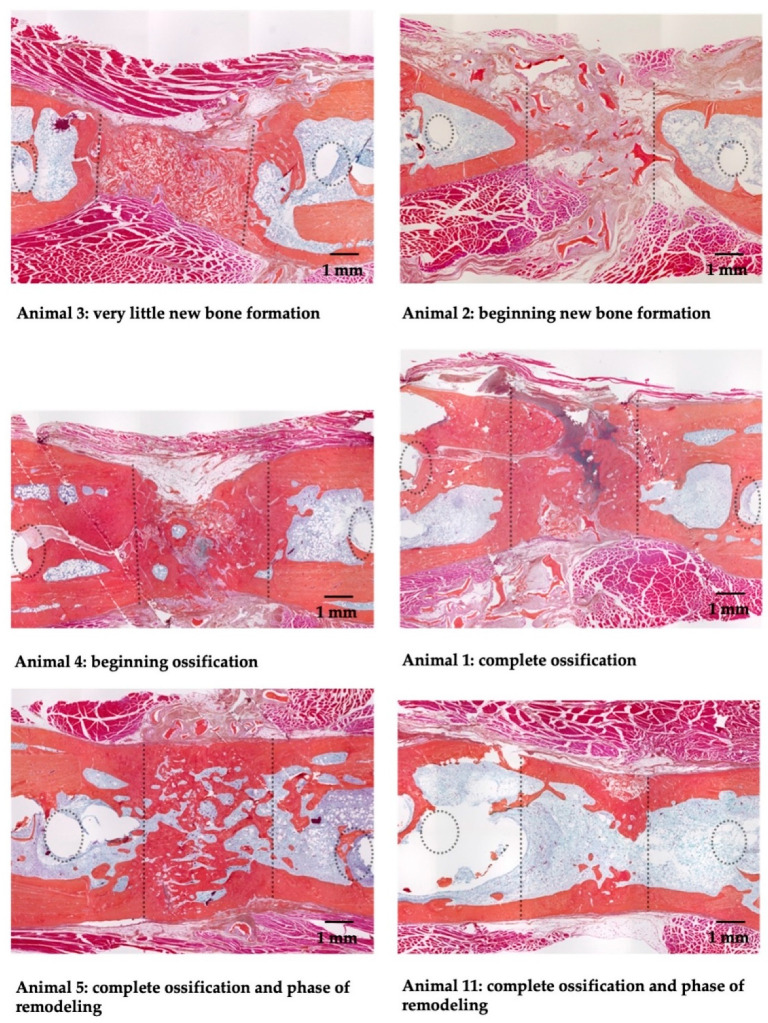
Histology of implantation site: Orange G+ Staining of the osseous defect zone. Bone defects were filled with cell-free f-DBM and were followed up for 2 months. The dotted lines show the original defect boundaries; the dotted circles the position of the screws closest to the defect.

Figure 3 shows histological Orange G+ staining of the defect area of all operated animals. A leukocytic infiltration of the tissue was not observed. 

All animals following surgery and f-DBM implantation exhibited new bone formation (Orange G+ staining). Two of the six animals showed complete bone healing with an already completed medullary cavity and a clearly advanced callus remodeling. In two animals the former osseus defect showed continuous callus formation, one defect was not completely bridged (the defect gap, however, almost closed). In two bone defects, only isolated, island-like bone formation could be observed. All animals of the control group had normal femur bones. Bone healing scores for all animals were shown in Table 1. The animal with ID 5 achieved the highest score with 37 points, the animal with ID 3 the lowest with 12. The results of the scoring indicate the beginning of bone formation also in the animals with a low score. Correlation analyses between the bone healing score and the measured blood parameters did not show a significant result for any of the measured parameters (*p* > 0.05).

### 3.3. Liver, Spleen, Kidney

**Figure 4:**
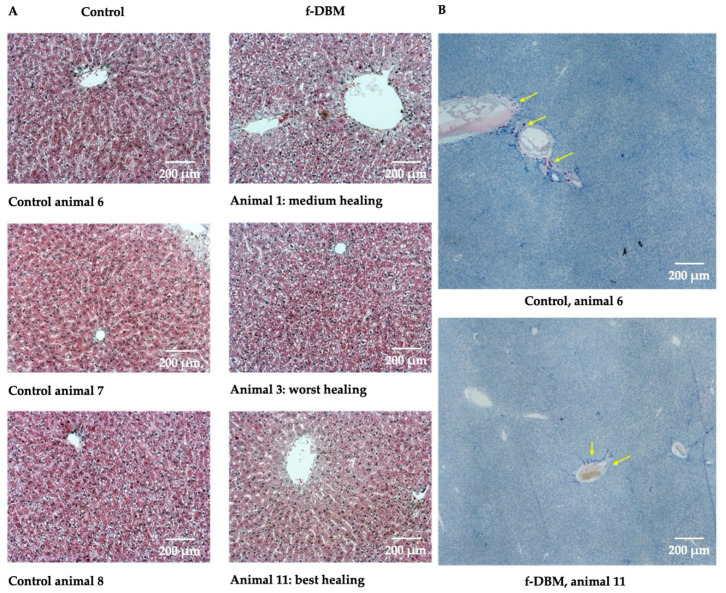

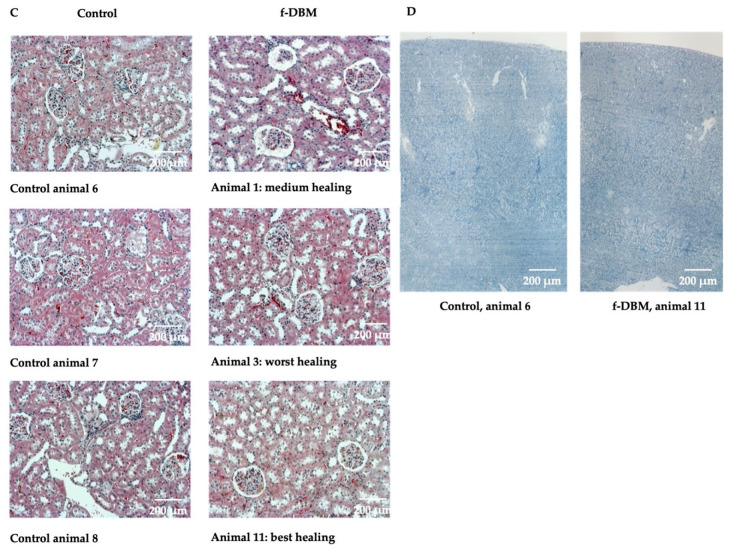
Histology of liver and kidney of all animals, that underwent surgery and untreated control. Histological analysis of the liver. (**A**) HE staining, organs of three control animals (left column) and three operated animals with different healing progressions (right column) are shown. In (**B**), chloroacetate esterase (CAE)-stained liver preparations from a control animal (top) and an operated animal (bottom) are shown. Yellow arrows mark leukocytes grouped around a blood vessel. This was equally common in both groups. In general, no correlation was observed between the healing process and the fine structure of the liver or leukocyte infiltration. Histological analysis of the kidney. (**C**) HE staining, organs of three control animals (left column) and three operated animals with different healing progressions (right column) are shown. In (**D**), CAE-stained kidney preparations from a control animal (left) and an operated animal (right) are shown. There was no leukocyte infiltration in any of the groups. In general, no correlation was observed between the healing process and the fine structure of the kidney or leukocyte infiltration.

Liver, spleen and kidney were also examined histologically. The focus was on signs of infectious infiltration (leukocytic invasion) and neoplastic events (abnormal cells). In CAE-stained liver preparations from control animals and operated animals leukocytes grouped around a blood vessel were equally common in both groups. In general, no correlation was observed between the healing process and the fine structure of the liver or leukocyte infiltration (Figure 4A,B). In histological analysis of the kidney, there was no leukocyte infiltration in any of the groups. In general, no correlation was observed between the healing process and the fine structure of the kidney or leukocyte infiltration (Figure 4C,D).

Histo-morphological data displayed no evidence of excessive leukocytic infiltration in any of the three organs compared to the unoperated control group.

### 3.4. Hematology 

The erythrocyte concentration, hemoglobin content and leukocyte concentration showed no differences between f-DBM and control group (Table 2). However, a significant shift within the standard range in the subpopulation ratio of the leukocyte fraction was detected. Thus, operated animals showed an increase in neutrophil content, while the lymphocyte fraction was decreased.

### 3.5. Serum Biochemistry

The sera of the operated animals showed significantly increased total protein concentrations. Creatinine, GOT, urea, glucose, GPT and ALP did not statistically differ from the serum parameters of control animals. It is noteworthy, that both groups had high glucose concentrations compared to the standard; however, the values were not measured sober (Table 3).

### 3.6. Free Radical Markers

Superoxid dismutase (SOD), malondialdehyde (MDA), glutathione (GSH) and nitrate/nitrite are part of a cell’s defence line against highly reactive molecules. An elevation suggests an exposure to harmful concentrations of free radicals. In this study, no significant rise of SOD, MDA, GSH or nitrate/nitrite was found in either group (Table 4).

### 3.7. Human HLA-Antibody Detection

Immune reaction against human HLA class I and class II antigens was detected with low mean fluorescence intensity (MFI) values equally in the serum of untreated and treated animals (Table 5). The antibodies against HLA class I antigens are, besides anti HLA-Cw12, which was detected in an untreated animal, reported as ‘natural antibodies’ that can also be found in nonalloimmunised healthy males and might be considered as false positive reactions of the serum due to denatured HLA-antigens on the beads [35]. Reactivities with clinically relevant MFI values ≥ 3000 were only found with these ‘natural antibodies’ in both treated and untreated animals.

**Figure 5:**
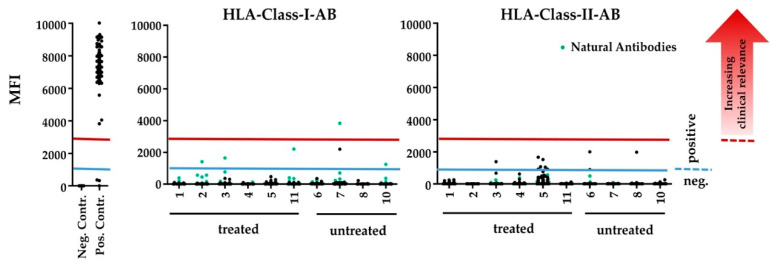
Reactivity against HLA-class-I- and HLA-class-II-antigens in the sera of untrated and treated animals. Each dot represents the reacitivty against a certain HLA-antigens. ‘Natural antibodies’ are marked with green dots. Antibodies with MFI ≥ 1000 were considered as positive (blue line), MFI-values ≥ 3000 were considered as clinically relevant immunization (red line).

Also, both HLA class I and class II antibodies, showed rather allele-specific reactions, than a conclusive pattern of reactivity in controls and treated animals. Thus, the reactivity against human HLA-antigens, detected in the serum of the rats reflects an unspecific background reaction rather than an immunisation by the human allografts (Table 5, Figure 5).

## 4. Discussion

In this study, we examined systemic toxicity, immunogenic behavior and osteogenicity of DBM fibers transplanted into a plate-stabilized, femoral critical-size-bone-defect of 5 mm in SD rats. The results do not suggest any systemic toxicity nor tumorigenicity of the bone substitute material. Furthermore, the operated animals showed no immune reaction to the examined bone graft, while histological examination mostly revealed significant bone healing.

### 4.1. Bone Growth

Study results on DBM use in bone defect treatment are discussed controversially [10,36,37]. Among manufacture-specific variations of different DBM batches, implantation site characteristics (size and character of the defect) and DBM properties (mechanical stability, solitary or composite use, cell-populated or not) must be taken into consideration to evaluate the osteogenic potential of DBM in situ [17,38]. Well vascularized and tight bone defects in close contact to vital bone allow DBM to develop its full potential [39,40]. To keep DBM concentrations high within the defect zone and to prevent ectopic bone formation, a *creeping-off* of the mostly low-viscous DBM paste must be avoided. Therefore, spatial separation is necessary. The clearly defined dental alveolar compartment provides these conditions. DBM paste exhibits significantly higher bone formation than synthetic bone replacement materials, if applied in the alveolar ridge [25]. Also, in cases of vertebral body fusion, freshly dissected intervertebral space offers optimized, osteoinductive conditions [21,23,26,37]. To evaluate DBM’s performance in critical size defects, it must first be distinguished between different defect models. Punch defects, in particular the rat calvarial defect model, are frequently used as experimental animal analogs for bone defects of critical size [36,37]. As seen before the defect offers a spatially restricted cavity, which ensures that the DBM preparation stays in place. However, a mechanical stimulus is missing and the studies on the osteogenic potential of DBM are contradictory [28]. Models of critical size defects in long tubular bones such as the femur can provide this mechanical stimulus through the movement of the animal [41,42,43]. Studies on the solitary application of solid DBM in critical size defects of large bones are rare. Most DBM is used as an osteoinductive additive in composite materials or as carrier for osteogenic cells or osteogenic mediators such as BMP-2 [29,38,44]. Pure DBM, with its high collagen content, is not very resistant and is rarely used in defects with high mechanical stress [45]. In these bone healing models, solid DBM without additional supplements induced a good bone healing response [29,46,47]. Complete healing of large defects, such as detected with vital autologous cancellous bone, was not observed.

This is in contrast to the results of the DBM material examined here. Used as the solitary filling material and only stabilized with a plate osteosynthesis, 2 out of 6 cases histologically showed excellent bone healing after 2 months and advanced consolidation in 2 cases. Bone development took place at a very low level in one animal only. A correlation of the bone healing result with the investigated physiological, hematological/immunological and biochemical parameters showed not significance. Furthermore, the absence of immigrated immune cells in the bone defect indicates that at least at the time of killing there was no relevant inflammatory response in the defect area. However, this observation does not rule out an individually different inflammatory reaction in the early phase of defect healing. There is evidence in the literature that the extent of initial inflammation can have significant effects on the bone healing outcome [48,49]. Too much inflammation causes the formation of fibrotic tissue in the defect, which inhibits the bony bridging of the defect. 

Since this work focused on the toxicological characteristics of the novel DBM, the control group consisted out of nonoperated animals. This is one limitation of this work regarding bone healing results. A significant comparison cannot be made with the control group. Nevertheless, the healthy, unoperated control group in this experiment serves the important comparison with the healthy animal, which is essential for HLA evaluations. The animals were kept under the same conditions, but received no surgery. A direct comparison of bone healing to the control group is therefore not possible. However, these results are promising. Cell-free β-tricalcium phosphate-based bone graft substitutes show a significantly lower rate of new bone formation using the same bone defect model [29]. With its coherent cotton-like structure, the material has a high surface/volume ratio. Studies have shown that the size of the DBM particles used and the surface/volume ratio have a significant influence on the osteogenic potential [50]. The large surface area of the fibrous DBM used in the present study allows for enhanced cell adherence and easier access to growth factors contained in the DBM. In addition to the mechanical factors already mentioned above, this could further support bone healing. Our data—compared to previous results in the same model—suggest improved bone healing when using fibrous DBM. However, an appropriately designed study is needed to support this assumption. 

DBM consists mainly of a collagen matrix with proteins and mineral residues [21]. Implanted into a living organism, rapid absorption within 6–8 weeks is expected. Lee et al., however, observed incorporation of the DBM into the reshaped bone [51]. At best, a small part was absorbed. This coincides with our own observations, in which DBM was still detectable after 8 weeks [29].

In the present case, the histological examinations were performed after 2 months. In all operated animals, whether with incomplete or complete ossification or even remodeling, only few DBM fibers were detected after this period. There are two possible explanations: either the DBM was completely resorbed, or no distinction between new bone and DBM residuals was possible after complete incorporation of the bone substitute.

### 4.2. Biocompatibility, Tumorigenicity and Immunogenicity

Since the development of DBM by Urist et al. in the 1970s, its high biocompatibility has been proven in a large number of animal and human studies [9,15,45,52,53]. These results were confirmed in this study. Clinically, there were no signs of foreign body reaction or rejection of the examined material (swelling, increased temperature, abscess formation, etc.). All surgical scars were inconspicuous. The behavior of the operated animals did not differ from the control group. But the body weight of the operated animals was significantly increased which is probably attributed to a decreased activity of the operated animals due to the opioid tramadol used for postoperative analgesia. Tramadol was described to persistently attenuate the movement activity of rats [54]. In addition, the added bone defect may cause the animal to relieve the limb, resulting in less movement and consecutive weight gain. Organ weights tend to be higher in operated animals, however the level of statistical significance was not reached. Presumably, increased formation of fat deposits occurred in all organs.

The high biocompatibility of the material is supported by the blood analyses. The measured serum level of creatinine, urea, GOT, GPT and ALP were not significantly elevated. This reflects a liver and kidney compatibility without toxic effects. The increased protein value in the f-DBM group was within the normal range for rats and could be explained by Tramadol-induced liver stress and/or a mild inflammatory response [55]. The increased glucose mobilization can be explained by stress during the preparations for the sacrifice. The concentrations of SOD, MDA, and GSH diverge to some extent from values measured in a similar study, also using SD rats [56]. The different analyte concentrations are most likely caused by conditional differences or lie in different evaluation strategies. The erythrocyte concentration and hemoglobin content did not differ significantly between the control group and the f-DBM group (Table 1). The concentration of white blood cells (WBC) per nanoliter is at a comparable level in both experimental groups. Further analysis shows a significant increase in neutrophil levels with a significant decrease in lymphocyte subpopulation in operated animals (Table 2). Correlation analyses indicates a nonsignificant, negative correlation between the healing result and the percentage of neutrophils (R = -0.46, p = 0.38). An accumulation of neutrophils in the tissue was not observed histologically, so that no clinical relevance seems to exist. In agreement with the laboratory analyses, no histological evidence of damage to liver (GOT, GPT) and kidney (creatinine, urea) could be observed. There were no signs of cholestasis (leading to accumulation of bile pigments) and fatty liver (cytoplasmic fat droplets, indirect detection in HE staining). Necrotic areas did not occur in either group (Figure 3). Leukocyte infiltrates in the immediate vicinity of vessels were little and found to be comparably common in both groups (via CAE staining, Figure 3).

The histological renal sections did not show any fine-structure difference in the glomeruli, tubules in the bark area, tubules in the medullary area and the papilla. In contrast to the liver, leukocyte infiltrates were not observed. There was no evidence of tumor formation in any of the histological examinations.

The use of DBM is considered safe [15]. No reports of serious immune reactions or rejection can be found. The most important argument for its safety is the controlled conditions DBM is manufactured in. The manufacturing process completely removes viable cells from the osseous raw material. A collagen scaffold with proteins, mineral residues and a small amount of cell debris remains [15,57]. Nevertheless, the conditions ought to be chosen so that the osteogenic proteins are not destroyed. The presence of residues of immunogenic cell components and proteins is possible. Thus, Fretwurst et al. reported the detection of HLA I and HLA II proteins in processed allogenic bone blocks of bigger size [13]. It is suggested that they possibly trigger immune reactions, leading to foreign body reactions and implant failure.

In this study, we tested whether rats treated with allogenic bone demonstrate a specific immune reaction. Our focus was on antibodies against human MHC I and II in the treated rat blood. An immune response against these two antigens could have been expected if the cell removal process was not effective. A possible direct immunogenicity with human collagen as the main component cannot be detected by the HLA determination in the used rat model. However, there is a very high interspecies homology of collagens so that the results can be transferred very well [58]. In addition, no adverse or immune reactions have been reported in human clinical trials with injected collagen I and III [59]. Only an unspecific background reaction with rather low MFI-values without significant immunization patterns were found in both untreated and treated animals, when the sera of the rats were tested for anti-human HLA antibodies. MFI values of over 3000 are considered a positive predictive factor for human kidney transplant rejection [60]. Such high MFi-values could only be measured in an untreated animal in context with a ‘natural antibody’ and was therefore considered as unspecific reaction Thus, an immunization against human HLA antigens by the allograft could not be confirmed.

## 5. Conclusions

In the present study, a potential toxicity and immunogenic effects of f-DBM allograft in a rat femoral defect model compared to nonoperated animals was analyzed after 2 months on the basis of blood and organ samples in addition to the assessment of bone healing. The analyses of blood and organs did not show any relevant clinical signs of toxicity of f-DBM. Biochemical, hematological and histological analyses remained without findings in comparison to the untreated control group. Increased/decreased values (neutrophil percentage, lymphocyte percentage, protein concentration) in the treated group are within normal range. The analysis of free radical biomarkers revealed no difference between the two experimental groups. No evidence for an immunization of the host was found. The entirety of the biochemical findings in combination with the histological findings of the liver and kidney confirm that f-DBM is nontoxic and does not lead to host-sensitization. Interestingly, the fibrous DBM preparation investigated here displayed a remarkable osteogenic effect, calling for further confirmatory studies.

## Figures and Tables

**Table 1:** Bone Healing Score as described in Material and Methods; the animal ID was assigned for evaluation and is kept constant throughout the document.

ID	Bone Healing Score
1	28
2	14
3	12
4	20
5	37
11	33

**Table 2:** Hematological results of both groups: Significantly increased percentage of neutrophilic granulocytes, significantly reduced percentage of lymphocytes in operated animals after two months of standing. Standard ranges by Delwatta et al. [34].

Haematological Marker	Control (Median)	f-DBM (Median)	Statistics	Standard Range
Erythrocytes [cells/pL]	6.4	6.3	p = 0.26	3.8–6.7
Hemoglobin [g/dL]	15.6	15.8	p = 0.9	10.4–16.5
White blood cells [cells/nL]	13.35	12.85	p = 0.71	4.4–14.8
Lymphocytes [%]	85.5	81.3	p = 0.03	61–86
Neutrophiles [%]	13.4	16.6	p = 0.03	13–36
Monocytes [%]	0.94	0.98	p = 0.71	0–1

**Table 3:** Results of serum biochemistry of both groups: Significantly increased total protein concentrations in f-DBM animals. Standard ranges by Delwatta et al. [34]. * Sober blood sugar range.

Blood Serum Marker	Control (Median)	f-DBM (Median)	Statistics	Standard Range
Creatine [mg/dL]	0.37	0.46	p = 0.47	0.2–0.8
Urea [mg/dL]	39	36.5	p = 0.48	32–53
Protein [g/dL]	5.4	6.5	p = 0.04	5.6–7.6
Glucose [mg/dL]	294	245	p = 0.07	62–202 *
GOT/AST [U/L]	155	192	p = 0.35	0.2–838
GPT/ALT [U/L]	39	38	p = 0.26	1–223
ALP [U/L]	156	148	p = 0.91	160–838

**Table 4:** Load of free radicals. At the age of two months, no significant differences were observed between the groups. SOD = superoxide dismutase, MDA = malondialdehyde, GSH = glutathione.

Free Radical Marker	Control (Median)	f-DBM (Median)	Statistics
SOD [U/mL]	0.29	0.27	p = 0.47
MDA [µmol/L]	10.59	10.1	p = 0.48
GSH [µmol/L]	12.62	19.56	p = 0.25
Nitrate/Nitrite [µmol/L]	22.6	34.61	p = 0.26

**Table 5:** Results of HLA measurements after two months. Results are shown in MFI, values greater 1000 were rated positive.

ID	Treatment	Anti HLA Class I [MFI]	Anti HLA Class II [MFI]
6	control	neg	DQ5 (DQB1*05:01) [1993]
7	control	A80 [3831]; Cw12 [2196]	neg
8	control	neg	DQA1*03:03 [1973]
10	control	A11 (A*11:02) [1239]	neg
1	f-DBM	neg	neg
2	f-DBM	A23 [1414]	neg
3	f-DBM	A30 (A*30:01) [1643]	DP13 [1387]
4	f-DBM	neg	neg
5	f-DBM	neg	DR4 (DRB1*04:05) [1662]; DR14 (DRB1*14:01 *14:54) [1516]; DQ2 [1066]
11	f-DBM	A43 [2209]	neg
